# Prevalence and clinical relevance of interview-assessed psychosis-risk symptoms in the young adult community

**DOI:** 10.1017/S0033291717002586

**Published:** 2017-09-11

**Authors:** Frauke Schultze-Lutter, Chantal Michel, Stephan Ruhrmann, Benno G. Schimmelmann

**Affiliations:** 1 University Hospital of Child and Adolescent Psychiatry and Psychotherapy, University of Bern, Bern, Switzerland; 2 Department of Psychiatry and Psychotherapy, Medical Faculty, Heinrich-Heine University, Düsseldorf, Germany; 3 Developmental Clinical Psychology Research Unit, Faculty of Psychology and Educational Sciences, University of Geneva, Geneva, Switzerland; 4 Department of Psychiatry and Psychotherapy, University of Cologne, Cologne, Germany; 5 University Hospital of Child and Adolescent Psychiatry, University Hospital Hamburg-Eppendorf, Hamburg, Germany

**Keywords:** Basic symptoms, clinical relevance, general population, prevalence, psychoses, ultra-high risk

## Abstract

**Background:**

An efficient indicated prevention of psychotic disorders requires valid risk criteria that work in both clinical and community samples. Yet, ultra-high risk and basic symptom criteria were recently recommended for use in clinical samples only. Their use in the community was discouraged for lack of knowledge about their prevalence, clinical relevance and risk factors in non-clinical, community settings when validly assessed with the same instruments used in the clinic.

**Methods:**

Using semi-structured telephone interviews with established psychosis-risk instruments, we studied the prevalence of psychosis-risk symptoms and criteria, their clinical relevance (using presence of a non-psychotic mental disorder or of functional deficits as proxy measures) and their risk factors in a random, representative young adult community sample (*N*=2683; age 16–40 years; response rate: 63.4%).

**Results:**

The point-prevalence of psychosis-risk symptoms was 13.8%. As these mostly occurred too infrequent to meet frequency requirements of psychosis-risk criteria, only 2.4% of participants met psychosis-risk criteria. A stepwise relationship underlay the association of ultra-high risk and basic symptoms with proxy measures of clinical relevance, this being most significant when both occurred together. In line with models of their formation, basic symptoms were selectively associated with age, ultra-high risk symptoms with traumatic events and lifetime substance misuse.

**Conclusions:**

Psychosis-risk criteria were uncommon, indicating little risk of falsely labelling individuals from the community at-risk for psychosis. Besides, both psychosis-risk symptoms and criteria seem to possess sufficient clinical relevance to warrant their broader attention in clinical practice, especially if ultra-high risk and basic symptoms occur together.

## Introduction

Psychotic disorders are frequently chronic disorders causing severe disability; thus, incurring high direct and indirect costs and psychosocial burden (Gustavsson *et al.* 2011). Oftentimes, significant delays in the initiation of adequate treatment contribute to poor outcome (Penttilä *et al.* 2014), which are fostered by stigmatising, negative attitudes both towards people with mental illness and towards help-seeking for mental problems (Schnyder *et al.* 2017). Stigma against people with mental illness, in turn, is primarily fuelled by illness-associated unusual behaviours that, in particular in case of psychoses, are perceived by others as unpredictable and dangerous (Corcoran, 2016; Imhoff, 2016). Thus, a comprehensive early detection of and intervention in persons at increased risk for developing a psychotic disorder may not only improve outcomes and reduce costs (Fusar-Poli *et al.* 2013) but may also reduce stigmatization by avoiding overt psychotic symptoms and the label ‘schizophrenia’, and by providing adequate education early on (Corcoran, 2016; Imhoff, 2016). Yet, as only few persons with a beginning psychosis seek help in their prodromal phase (Schultze-Lutter *et al.* 2015*a*; Kazdin, 2017), a comprehensive preventive approach would require assertive community programs, incl. effective outreach screening and awareness programs, in order to reduce significantly the incidence of psychosis at community level. These, in turn, require good knowledge about the prevalence and clinical relevance of the presumed psychosis-risk symptoms and criteria in the community.

The two complementary current approaches to a psychosis-risk detection comprise: (1) the three ultra-high risk criteria whose two symptomatic criteria include mainly attenuated (APS) but also brief intermittent psychotic symptoms (BIPS), and (2) the two basic symptom criteria, cognitive–perceptive basic symptoms (COPER) and cognitive disturbances (COGDIS) that mainly include subjective cognitive disturbances (Fusar-Poli *et al.* 2013; Schultze-Lutter *et al.* 2015*b*). Supplementary Text S1 provides details on psychosis-risk approaches and criteria. Recently, the European Psychiatric Association recommended the APS and BIPS criteria and COGDIS for alternative use in psychosis-risk detection (Schultze-Lutter *et al.* 2015*b*). The genetic risk-functional decline criterion of the ultra-high risk approach was not recommended for lack of evidence of a relevant risk enhancement, COPER was not recommended for lack of research on it. Furthermore, restricting the use of psychosis-risk criteria to individuals distressed by mental problems and seeking help for them was recommended. Any clinical screening of other individuals was regarded as not warranted by current scientific evidence for the lack of studies outside clinical settings (Schultze-Lutter *et al.* 2015*b*).

So far, community studies in representative samples exclusively targeted presumed APS/BIPS, never basic symptoms. With two exceptions (Schultze-Lutter *et al.* 2014*a*; Schimmelmann *et al.* 2015), these have never used special instruments for psychosis-risk assessment, although some clinician-assessed interview-studies have been conducted in selected, often child and adolescent samples using assessments for psychotic symptoms (Spauwen *et al.* 2003, 2006; Hanssen *et al.* 2005; Kelleher *et al.* 2012*a*, *b*; Asher *et al.* 2013; Nuevo *et al.* 2013; Jeppesen *et al.* 2015). The majority of community studies on alleged psychotic experiences, however, was conducted with self-report questionnaires or fully standardized layperson interviews. From these, a median prevalence of 7.2% of so-called ‘psychotic-like experiences’ was estimated (Linscott & van Os, 2013). These psychotic-like experiences were frequently assumed to resemble APS (Schultze-Lutter *et al.* 2011, 2014*b*), although the mode of assessment accounted for most of the variance (19.7%) in the observed rates, indicating a great overestimation of psychotic-like experiences by questionnaires (Linscott & van Os, 2013; Schultze-Lutter *et al.* 2014*b*). Additionally, beside sociodemographic risk factors for the presence of psychotic-like experiences, higher rates were also observed in convenience, and non-dispersed and smaller samples (Linscott & van Os, 2013).

### Aims of the study

To close the gap of knowledge on psychosis-risk symptoms and criteria in the community when validly assessed in accordance with their assessment in clinical samples, we studied their point-prevalence and clinical relevance as well as risk factors for their presence in a large, random, representative general population sample of young adults. For the reported higher psychosis-predictive power of the combined presence of ultra-high risk and basic symptoms (Fusar-Poli *et al.* 2016), we expected the highest clinical relevance for this combination. Additionally, for their conceptualization as the most immediate psychopathological manifestation of neurobiological aberrations underlying psychoses (Schultze-Lutter *et al.* 2016), we expected basic symptoms to be most strongly associated with risk factors related to neurobiology, such as genetic vulnerability and age.

## Methods

### Study design

The Bern Epidemiological At-Risk (BEAR) study used a stratified sampling method to obtain a representative sample of 7370 people aged 16–40 years from the approximately 310 000 predominantly Caucasian people of this age registered in the semi-rural Canton of Bern, whose largest city has about 134 000 citizens; 21% of its population is non-Swiss (80% from European countries). The age range of 16–40 years was selected because most first episodes of affective and non-affective psychoses and psychotic symptoms (interquartile range of the 25^th^ and 75^th^ percentiles) are reported to occur between 17 and 41 years of age (Kirkbride *et al.* 2006; McGrath *et al.* 2016). Stratified by sex (1:1), potential participants were randomly drawn from the population register including their address, date of birth, sex, nationality, and parents’ names (for minors). Telephone numbers were subsequently searched in directories and the Internet. The ethics committee of the University of Bern approved the study; participation in the telephone interview indicated that informed consent had been provided.

### Procedure

Recruitment and assessments were conducted over 3.5 years (June 2011–November 2014) supported by the Computer-Assisted Telephone Interviewing technique. Prior to commencing the study, a feasibility study of the reliability of telephone assessments of psychosis-risk symptoms and criteria in comparison with the gold standard of face-to-face assessments found excellent concordance rates of 86–100% (Michel *et al.* 2014).

To increase the response rate, the first contact was established by sending a one-page information letter to potential participants and, if minors, to their parents. The letter explained the aims of the study, voluntariness of participation, participation-associated lottery, data security and anonymity, and non-report of findings to avoid violating the ‘right not to know’ (Koponen & Aromaa, 2017). First telephone contact was attempted within 2 weeks of sending the letter. The lottery with monetary winnings (40–2000 CHF) at an announced 1:50 chance of winning served as an incentive to counteract the known bias in epidemiological studies towards individuals with a higher educational background and high interest in the study's topic (Guyll *et al.* 2003).

### Participants

In addition to age range and main residency (i.e. a valid address and not being abroad during the assessment period) in the Canton of Bern, an available telephone number was required for eligibility. Interviews were aborted prematurely when it became clear that respondents had a lifetime diagnosis of psychosis or insufficient language skills in German, French, English, or Spanish. Telephone numbers not answered in 100 attempts made at various times and days, including Saturdays, over several months were considered suggestive of long-time absence and, consequently, of unknown eligibility.

### Assessments

#### Assessment of psychosis-risk symptoms and criteria

Psychosis-risk symptoms and criteria (for further details, see online Supplementary Text S1) were assessed for lifetime presence, onset, and current frequency using two semi-structured instruments for that good interrater reliability between trained raters has been reported (Schultze-Lutter *et al.* 2007; McGlashan *et al.* 2010):

• The Structured Interview for Psychosis-Risk Syndromes (McGlashan *et al.* 2010), a main instrument for assessing ultra-high risk criteria (Schultze-Lutter *et al.* 2013), in brief, defines the APS criterion by (1) at least one of the five positive items with a score on the seven-point Likert scale of ‘3’ (moderate) to ‘5’ (severe but not psychotic), (2) first occurrence or worsening within the past 12 months, and (3) at least weekly occurrence within the past month. The BIPS criterion is defined by (1) at least one of these five positive items with a score of ‘6’ (severe and psychotic), which (2) was reached within the past 3 months and (3) was present at least for several minutes per day at a frequency of at least once per month. APS and BIPS were only rated if the phenomenon in question was not fully and better explained by another non-psychotic disorder or an effect of psychotropic drug use (McGlashan *et al.* 2010; Schultze-Lutter *et al.* 2013).
  The genetic risk-functional decline criterion was estimated only with a first-degree relative of psychosis serving as a genetic risk factor and being assessed with the Structured Interview for Psychosis-Risk Syndromes; schizotypal personality disorder was not assessed because of the lack of an informant (Tyrer *et al.* 2007).
• The Schizophrenia Proneness Instrument, Adult version (Schultze-Lutter *et al.* 2007), used for assessing basic symptom criteria in adults defines COPER by (1) at least one of ten basic symptoms with (2) first occurrence at least 12 months ago and (3) an occurrence of at least ‘several times in a month or weekly’ within the past 3 months (Schultze-Lutter *et al.* 2015*b*). COGDIS requires (1) at least any two of nine cognitive basic symptoms of that five are also included in COPER with (2) an occurrence of at least ‘several times in a month or weekly’ within the past 3 months (Schultze-Lutter *et al.* 2015*b*). Strictly, the definition of basic symptoms includes the requirement that the phenomenon in question presents a deviation from the ‘normal’ self. Nevertheless, to allow the rating of lifelong persistent complaints, the Schizophrenia Proneness Instrument, Adult version also includes a rating of ‘7’, ‘has always been present in the same severity (trait)’.

#### Assessment of mental disorders

The presence of DSM-IV axis-I disorders, which can be validly assessed on the telephone (Rohde *et al.* 1997), was assessed using the Mini-International Neuropsychiatric Interview (Sheehan *et al.* 1998). In combination with the Structured Interview of Psychosis-Risk Syndromes, it was also used to assess past and present psychoses and their type as part of the exclusion criteria (for details on this group see Michel *et al.* 2016). Requiring about 25% of the assessment time of other scales for the assessment of axis-I disorders, the Mini-International Neuropsychiatric Interview possesses good construct validity with other established scales and expert diagnoses as well as good interrater and retest reliability (Sheehan *et al.* 1998). Furthermore, it has been successfully applied in telephone interviews with non-clinical samples (Wang *et al.* 2006).

#### Assessment of functioning

Psychosocial functioning was estimated using the Social and Occupational Functioning Assessment Scale (APA, 1994). A score ⩽70 was regarded as indicative of a functional deficit (Schimmelmann *et al.* 2015).

#### Quality assurance

To achieve a ⩾95% concordance rate with the trainers (F.S.-L. and C.M.), interviewers (all clinical psychologists) received intensive 3-month training, especially in the semi-structured context-dependent personalized assessment of psychosis-risk symptoms and mental disorders. In line with clinical assessments, this routinely included gathering thorough information on:

• situations in that the phenomenon had occurred,
• the degree of externalization / conviction,
• participant's reaction in response to / explanation of the potential symptom incl. distress,
• reactions of others (in particular, others’ opinion on potential ‘unusual thought content’ to control for ‘normal’ subcultural believes),
• potential associations with substance use, somatic / known neurological conditions or hypnagogic/hypnopompic states.

Additionally, weekly supervisions of all symptom ratings in case of conferences with the interviewers on the basis of all available information performed by either of two very experienced experts in the early detection of psychosis (F.S.-L. or C.M.) ensured excellent, valid and reliable data quality.

### Statistical analyses

Using Statistical Package for Social Sciences v23, the frequencies and percentages were compared using χ^2^ tests and non-normally distributed continuous and ordinal data using Mann–Whitney *U* tests and the respective effect sizes. The associations of functioning and mental disorder, as well as of the potential risk factors with current psychosis-risk symptoms and criteria were explored by binary and multinomial regression analyses using the Omnibus test as a goodness-of-fit measure. Stepwise regression analyses were performed forward and backward to test for the model stability.

## Results

### Recruitment and representativeness of sample

**Fig. 1.**
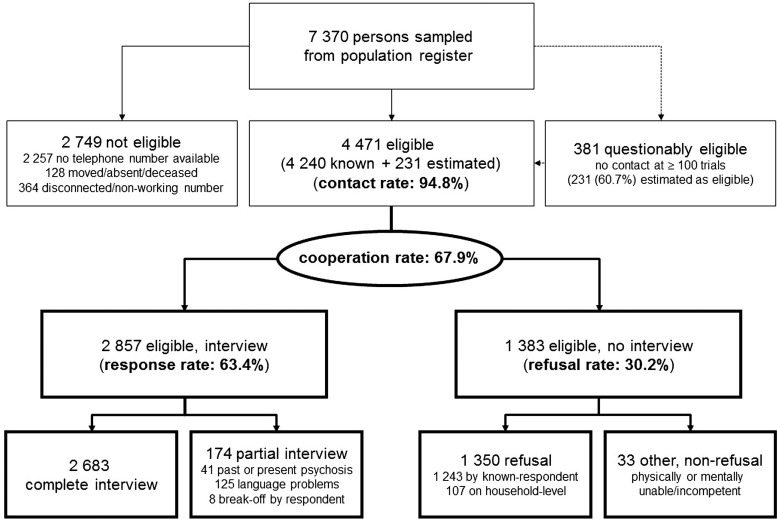
Results of recruitment. Survey outcome rates of the BEAR study according to the definitions of the American Association for Public Opinion Research (AAPOR, 2016).

Of the initial sample (*N* = 7370), 4471 were eligible (Fig. 1). The contact rate was 94.8% and the response rate 63.4%. Of the 2857 interviews, 125 (4.4%) were aborted prematurely by interviewers for insufficient language skills and 41 (1.4%) for lifetime psychosis (Michel *et al.* 2016). Only eight (0.6%) participants terminated the interview of their own accord; 2683 (93.9%) interviews were completed, which took 43 min on average (standard deviation: 20 min). Almost all participants considered the interview as very or rather pleasant (97.9%) and not stressful (97.5%); 97.9% agreed to be re-contacted for a similar interview in future.

The eligible sample was slightly older than the 16- to 40-year-old general population of Bern, with an extremely small effect size of d = 0.053. Yet, no age group was significantly over- or underrepresented (Table 1).

**Table 1.** Estimations of the representativeness of the study sample at various levels of recruitment

Comparison of the eligible sample with the Canton Bern general population according to the Swiss Statistics Web site for 2014, maintained by the Federal Statistical Office (http://www.bfs.admin.ch)			
	Canton Bern (*N* = 3 10 708)	Eligible sample (*N* = 4 471)	Statistics^a^
Age (mean ± standard deviation)	27.1 ± 7.1 years	30.3 ± 7.2 years	t_(315177)_ = 29.916, *p* < 0.001, d = 0.053
Age ranges (%)			
16–20 years	16.9	14.6	χ^2^_(1)_ = 0.168, *p* = 0.682, *w* < 0.001
21–25 years	19.4	16.7	χ^2^_(1)_ = 0.202, *p* = 0.653, *w* < 0.001
26–30 years	21.0	12.5	χ^2^_(1)_ = 2.157, *p* = 0.142, *w* = 0.003
31–35 years	21.8	21.4	χ^2^_(1)_ = 0.004, *p* = 0.950, *w* < 0.001
36–40 years	20.9	34.6	χ^2^_(1)_ = 3.382, *p* = 0.066, *w* = 0.003
Sex; % male	50.3	55.6	χ^2^_(1)_ = 0.265, *p* = 0.607, *w* < 0.001
Nationality; % Swiss	78.8	91.6	χ^2^_(1)_ = 0.962, *p* = 0.327, *w* = 0.002
Comparison of the participants and refusers			
	Participants (*N* = 2857)	Refusers (*N* = 1350)	Statistics^b^
Age (mean ± standard deviation)	30.3 ± 7.5 years	30.9 ± 7.4 years	*U* = 1 832 908.5 *p* = 0.009, *r* = 0.040
Sex; % male	54.1	57.6	χ^2^_(1)_ = 4.678, *p* = 0.031, V = 0.033^b^
Nationality; % Swiss	91.1	92.2	χ^2^_(1)_ = 3.946, *p* = 0.047, *V* = 0.031^b^
Comparison of the persons with a complete and a partial interview			
	Complete (*N* = 2683)	Partial (*N* = 174)	Statistics^b^

Age (mean ± standard deviation)	30.2 ± 7.6 years	32.1 ± 6.3 years	*U* = 205 488.0, *p* = 0.008, r = 0.050
Sex; % male	54.0	55.7	χ^2^_(1)_ = 0.208, *p* = 0.648, *V* = 0.009
Nationality; % Swiss	93.6	51.7^c^	χ^2^_(1)_ = 352.948, *p* < 0.001, *V* = 0.351
Comparison of participants with a complete interview with the Canton Bern general population according to the Swiss Statistics Web site for 2014, maintained by the Federal Statistical Office			
	Canton Bern (N = 3 10 708)	Complete (N = 2 683)	Statistics^a^
Age (mean ± standard deviation)	27.1 ± 7.1 years	30.2 ± 7.6 years	t_(313389)_ = 22.505, *p* < 0.001, *d* = 0.040
Age ranges (%)			
16–20 years	16.9	16.1	χ^2^_(1)_ = 0.019, *p* = 0.890, *w* < 0.001
21–25 years	19.4	16.5	χ^2^_(1)_ = 0.234, *p* = 0.629, *w* < 0.001
26–30 years	21.0	11.5	χ^2^_(1)_ = 2.777, *p* = 0.096, *w* = 0.003
31–35 years	21.8	20.9	χ^2^_(1)_ = 0.019, *p* = 0.890, *w* < 0.001
36–40 years	20.9	34.9	χ^2^_(1)_ = 3.513, *p* = 0.061, *w* = 0.003
Sex; % male	50.3	54.0	χ^2^_(1)_ = 0.131, *p* = 0.717, *w* < 0.001
Nationality; % Swiss	78.8	93.6	χ^2^_(1)_ = 1.271, *p* = 0.260, *w* = 0.002
Marital status (%)			
Single	66.9	55.9	χ^2^_(1)_ = 0.985, *p* = 0.321, *w* = 0.002
Married/cohabitation	30.4	40.4	χ^2^_(1)_ = 1.412, *p* = 0.235, *w* = 0.002
Separated/divorced/widowed	2.7	3.7	χ^2^_(1)_ = 0.156, *p* = 0.693, *w* < 0.001

a Effect sizes were Cohen's *d* for the *t* test and the effect size index, *w*, for the one-dimensional χ^2^-tests.

b Effect sizes were Rosenthal's *r* for the Mann–Whitney *U* test and Cramer's *V* for χ^2^-tests.

For Cohen's *d*, *d* = 0.2 equals a small effect, *d* = 0.5 a medium effect, and d = 0.8 a large effect; for the effect size index *w*, Rosenthal's *r* and Cramer's *V*, 0.1 equals a small effect, 0.3 a medium effect, and 0.5 a large effect.

c Includes 125 (71.8%) participants with whom the interview has to be terminated prematurely for language reasons, all naturally non-Swiss participants.

The main reasons for refusal were a lack of interest or time (online Supplementary Table S2). Participants differed marginally from refusers in age, sex, and Swiss nationality; all differences were of extremely small effect size (Table 1). More interviews with non-Swiss individuals were aborted for language-related reasons. Additionally, participants who completed the interviews were slightly older than those who aborted interviews (Table 1). Similar to the observation in the eligibility sample, the 2683 participants differed marginally from the 16- to 40-year-old general population of Bern in mean age, but not in distribution across age groups, sex, nationality, or marital status (Table 1). Thus, as no response bias was detectable beyond the negligible age-related inclusion bias, participants were well representative of their age group. Their sample characteristics are provided online in Supplementary Table S3.

### Prevalence of psychosis-risk symptoms and criteria

In total 659 (24.6%) participants reported at least one lifetime psychosis-risk phenomenon; 460 (17.1%) had experienced one around the time of the interview. When trait-like phenomena (reported as always having been present at the same frequency and severity and, consequently, strictly not meeting the general requirement for a change in mental state) were excluded, the numbers went down to 567 (21.1%) for lifetime and 370 (13.8%) for current psychosis-risk symptoms (Table 2). Table 2 provides the prevalence rates of single symptoms.

**Table 2.** Prevalence of psychosis-risk symptoms, lifetime and current as well as lifetime and current excluding trait-like phenomena (No., % of whole sample, N = 2683)

	Lifetime (*n* = 659)	Current (*n* = 460)	lifetime, excl. traits (*n* = 567)	Current, excl. traits (*n* = 370)
Ultra-high risk symptoms				
unusual thought content/delusional ideas (P1)				
APS (score 3–5)z	134 (5.0)	93 (3.5)	105 (3.9)	68 (2.5)
BIPS (score 6)	1 (0.04)	1 (0.04)	0	0
suspiciousness/persecutory ideas (P2)				
APS (score 3–5)	55 (2.0)	45 (1.7)	44 (1.6)	36 (1.3)
BIPS (score 6)	0	0	0	0
Grandiosity (P3)				
APS (score 3–5)	8 (0.3)	6 (0.2)	6 (0.2)	4 (0.1)
BIPS (score 6)	0	0	0	0
Perceptual abnormalities/hallucinations (P4)				
APS (score 3–5)	198 (7.4)	87 (3.2)	163 (6.1)	68 (2*.5)*
BIPS (score 6)	9 (0.34)	2 (0.08)	9 (0.34)	2 (0.08)
Disorganized communication (P5)				
APS (score 3–5)	19 (0.7)	19 (0.7)	^a^	^a^
BIPS (score 6)	0	0	0	0
Any one APS	316 (11.8)	200 (7.5)	265 (9.9)	154 (5.7)
Any one BIPS	10 (0.37)	3 (0.11)	9 (0.34)	2 (0.08)
Basic symptoms				
Thought interference	31 (1.1)	^b^	22 (0.8)	12 (0.4)
Thought blockages	112 (4.2)	^b^	91 (3.4)	65 (2.4)
Thought pressure	46 (1.7)	^b^	42 (1.6)	28 (1.0)
Thought perseveration	11 (0.4)	^b^	7 (0.3)	3 (0.1)
Disturbance of receptive speech	6 (0.2)	^b^	5 (0.2)	4 (0.1)
Disturbance of expressive speech	55 (2.1)	^b^	47 (1.8)	42 (1.6)
Disturbances of abstract thinking	18 (0.6)	^b^	12 (0.4)	4 (0.1)
Inability to divide attention	22 (0.8)	^b^	10 (0.4)	7 (0.3)
Captivation of attention, etc.	42 (1.6)	^b^	32 (1.2)	17 (0.6)
Unstable ideas of reference	99 (3.7)	^b^	85 (3.2)	39 (1.5)
Derealization	56 (2.1)	^b^	51 (1.9)	22 (0.8)
Decreased ability to discriminate between ideas and perception, etc.	27 (1.0)	^b^	23 (0.9)	13 (0.5)
Visual perception disturbances	104 (3.9)	^b^	89 (3.3)	49 (1.8)
Acoustic perception disturbances	107 (4.0)	^b^	99 (3.7)	52 (1.9)
Any one basic symptom	478 (17.8)	^b^	413 (15.4)	264 (9.8)
Any one COPER symptom	416 (15.5)	^b^	369 (13.8)	222 (8.3)
Any one COGDIS symptom	320 (11.9)	^b^	263 (9.8)	169 (6.3)

APS: attenuated psychotic symptom; BIPS: brief intermittent psychotic symptom; COPER: ‘cognitive–perceptive basic symptoms’, COGDIS: ‘cognitive disturbances’.

a No information because of the primarily observation-based rating of communication during the interview and the lack of an informant report on any potential change in the participant's communication style.

b No information for basic symptoms, because basic symptoms (per definition a change in mental processes and, consequently, no trait) reported to occur in a trait-like manner were not assessed for current frequency (0 = not present in last 3 months to 6 = daily).

**Fig. 2.**
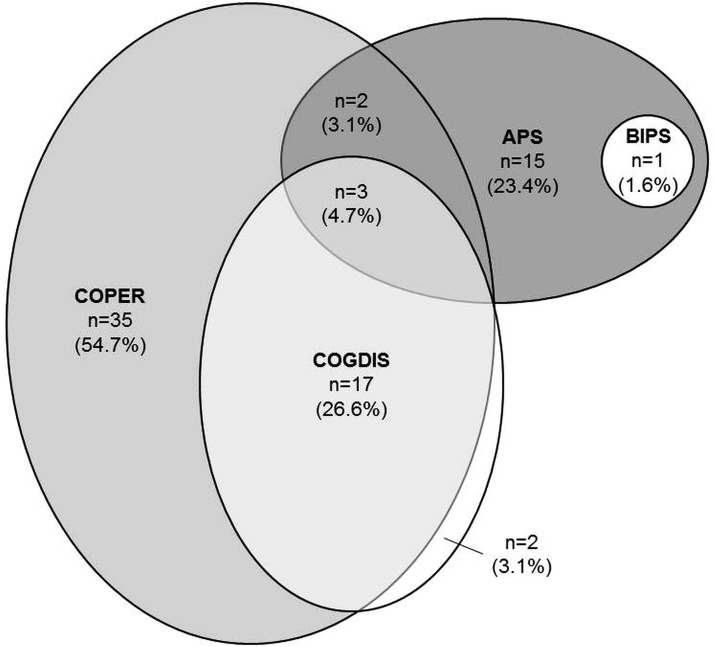
Distribution of psychosis-risk criteria (*n* = 64). APS: attenuated psychotic symptoms criterion; BIPS: brief intermittent psychotic symptoms criterion; COPER: cognitive–perceptive basic symptoms criterion; COGDIS: cognitive disturbances criterion. For detailed descriptions of criteria, see online Supplementary Text S1.

When the onset and frequency requirements of the psychosis-risk criteria were considered, altogether 64 (2.4%) participants met at least one criterion, most frequently COPER (*n* = 52, 1.94%; *n* = 15, 0.39%, exclusively) and never the genetic risk-functional decline criterion (Fig. 2). Only 29 (1.08%) met psychosis-risk criteria recommended by the European Psychiatric Association, i.e., APS, BIPS and/or COGDIS (Schultze-Lutter *et al.* 2015*b*). Five participants (0.19%) who met the APS criterion also met COPER and/or COGDIS (Fig. 2).

### Clinical relevance of psychosis-risk symptoms and criteria

Excluding trait-like phenomena, the presence of any current psychosis-risk symptom and any psychosis-risk criterion significantly predicted both the presence of any mental disorder and, more strongly, the presence of a functional deficit (Table 3). Taking into account the differential effects of APS/BIPS and basic symptoms, odds ratios (ORs) indicated the expected stepwise increase in the effects where the effect of the combined presence of ‘ultra-high risk and basic symptoms’ was the strongest on both symptom and criterion level (Table 3).

**Table 3.** Association of current psychosis-risk symptoms, excl. trait-like symptoms, and psychosis-risk criteria (entering as binary and multinomial variable, respectively) with presence of any non-psychotic axis-I DSM-IV disorder (n = 351) and presence of a functional deficit (SOFAS⩽70; n = 147)

	*β*	Standard error	Wald	df	*p* value^a^	Odds ratio	95% lower	CI upper
Presence of any non-psychotic mental disorder (*N* = 351)								
Any psychosis-risk symptom^b^ (*n* = 114)	1.361	0.132	10.607	1	< 0.001	3.901	3.013	5.051
Psychosis-risk symptoms^b,^^c^			117.007	3	<0.001			
Only basic symptoms (*n* = 54)	1.072	0.171	39.063	1	<0.001	2.920	2.087	4.086
Only APS/BIPS (n = 38)	1.588	0.214	55.168	1	<0.001	4.895	3.219	7.443
Both (*n* = 22)	2.003	0.298	45.277	1	<0.001	7.412	4.136	13.284
Any psychosis-risk criterion^b^ (*n* = 29)	1.777	0.258	47.404	1	<0.001	5.911	3.564	9.802
Psychosis-risk criteria,^c,^^d^			46.204	3	<0.001			
Only COPER/COGDIS (*n* = 21)	1.677	0.295	32.377	1	<0.001	5.350	3.002	9.534
Only APS/BIPS (n = 4)	1.559	0.648	5.787	1	0.016	4.756	1.335	16.943
Both (*n* = 4)	3.351	1.120	8.959	1	0.003	28.534	3.179	256.085
Presence of a functional deficit (*N* = 147)								
Any psychosis-risk symptom^b^ (n = 71)	1.948	0.176	122.200	1	<0.001	7.013	4.965	9.905
Psychosis-risk symptoms^b,^^c^			148.719	3	<0.001			
Only basic symptoms (*n* = 26)	1.399	0.239	34.099	1	<0.001	4.049	2.532	6.475
Only APS/BIPS (*n* = 27)	2.309	0.252	84.192	1	<0.001	10.060	6.144	16.472
Both (*n* = 18)	2.871	0.320	80.439	1	<0.001	17.661	9.430	33.076
Any psychosis-risk criterion^b^ (*n* = 29)	2.865	0.268	114.141	1	<0.001	17.554	10.378	29.695
Psychosis-risk criteria,^c,^^e^			113.698	3	<0.001			
Only COPER/COGDIS (*n* = 21)	2.766	0.304	82.954	1	<0.001	15.890	8.763	28.813
Only APS/BIPS (*n* = 5)	3.053	0.639	22.802	1	<0.001	21.186	6.050	74.190
Both (*n* = 3)	3.459	0.918	14.205	1	<0.001	31.780	5.260	192.005

Results of univariate logistic regression analyses.

APS, attenuated psychotic symptom; BIPS, brief intermittent psychotic symptom; COPER, cognitive–perceptive basic symptoms; COGDIS, cognitive disturbances; CI, confidence interval of odds ratio.

All models were highly significant with a goodness-of-fit of χ^2^_(1)_⩾41.075, *p*<0.001.

a When adjusting for multiple testing (four tests in each domain), the critical *p* value of each test is 0.0125.

b Correct prediction of absence of mental disorder/functional deficit: 100%; correct prediction of respective presence: 0%.

c Absence of any psychosis-risk symptom or criterion served as reference value.

d Correct prediction of absence of mental disorder: 100%; correct prediction of presence of mental disorder: 1.1%.

e Correct prediction of absence of functional deficit: 99.7%; correct prediction of presence of functional deficit: 5.4%.

### Risk factors for presence of psychosis-risk symptoms

The presence of any current non-trait-like psychosis-risk symptom was predicted by younger age, lifetime alcohol misuse, lifetime and current drug misuse, single marital status, no current partner, lower school education, unemployment, family history of mental disorders in first- or second-degree biological relatives (in particular of substance use and/or affective disorder), and lifetime traumatic events (Table 4). Sex, migrant status (estimated by non-Swiss nationality), minority status, current alcohol misuse, and higher population density did not predict the presence of any psychosis-risk symptom (Table 4). Stepwise analyses revealed a stable significant model (goodness-of-fit: χ^2^_(7)_ = 72.048, *p*<0.001) including younger age, lifetime drug misuse, no current partner, lower school education, unemployment, family history of mental disorders, and lifetime traumatic event as the predictors of any current psychosis-risk symptom (Table 4).

**Table 4.** Association of current non-trait-like psychosis-risk symptoms with predictors described for psychotic-like experiences, assessed by questionnaires or fully-standardized lay-person interviews for psychotic symptoms in the community (Linscott & van Os, 2013) (N = 2683)

Results of univariate logistic regression analyses							
	*β*	Standard error	Wald (df = 1)	*p* value	Odds ratio	95% lower	CI upper
Age^a^ (in years)	*−0.027*	*0.007*	*13.810*	*<0.001*	*0.973*	*0.959*	*0.987*
Male sex^b^	*−*0.183	0.112	2.666	0.103	0.833	0.669	1.037
School education^a^	*−0.162*	*0.076*	*4.521*	*0.033*	*0.851*	*0.733*	*0.987*
Current unemployment^a^	*0.801*	*0.296*	*7.330*	*0.007*	*2.227*	*1.247*	*3.977*
Migrant status^b^	*−*0.170	0.218	0.613	0.434	0.843	0.551	1.292
Minority status^b^	0.636	0.469	1.843	0.175	1.890	0.754	4.737
Single marital status^a^	*0.303*	*0.115*	*6.937*	*0.008*	*1.354*	*1.081*	*1.696*
No current partner^a^	*0.426*	*0.118*	*12.977*	*<0.001*	*1.531*	*1.214*	*1.931*
Family history of mental disorders^a^,^c^	*0.486*	*0.113*	*18.576*	*<0.001*	*1.626*	*1.303*	*2.028*
Lifetime traumatic event^a^	*0.599*	*0.158*	*14.418*	*<0.001*	*1.820*	*1.336*	*2.479*
Lifetime alcohol misuse^a^	*0.635*	*0.239*	*7.052*	*0.008*	*1.887*	*1.181*	*3.016*
Current alcohol misuse^b^	0.665	0.408	2.651	0.103	1.944	0.873	4.327
Lifetime drug misuse^a^	*0.634*	*0.177*	*12.775*	*<0.001*	*1.885*	*1.332*	*2.669*
Current drug misuse^a^	*0.971*	*0.348*	*7.785*	*0.005*	*2.640*	*1.335*	*5.221*
Population density (person/km^2^)^b^	0.000	0.000	0.999	0.317	1.000	1.000	1.000
Results of stepwise logistic regression analyses (Wald method, forward and backward)							
Age	*−*0.250	0.008	9.205	0.002	0.975	0.960	0.991
School education	*−*0.186	0.078	5.707	0.017	0.830	0.712	0.967
Current unemployment	0.637	0.304	4.380	0.036	1.890	1.041	3.432
No current partner	0.316	0.132	5.688	0.017	1.372	1.058	1.778
Family history of mental disorders	0.562	0.117	23.091	<0.001	1.754	1.395	2.206
Lifetime traumatic event	0.505	0.162	9.672	0.002	1.657	1.205	2.278
Lifetime drug misuse	0.505	0.182	7.692	0.006	1.658	1.160	2.369

CI, confidence interval of odds ratio.

a All models were significant with a goodness-of-fit of χ^2^_(1)_⩾4.589, *p*<0.005.

b All models were non-significant with a goodness-of-fit of χ^2^_(1)_⩽2.664, *p*>0.103.

c Any first- or second-degree biological relative with a mental disorder reported by the interviewee in the Structured Interview for Psychosis-Risk Syndromes.

Significant variables at a p-level of 5% in univariate analyses are displayed in *Italics*.

When ultra-high risk and basic symptoms were distinguished (online Supplementary Table S4), the following predictors of psychosis-risk symptom constellations emerged:

• exclusively ultra-high risk symptoms: family history of mental disorders, lifetime trauma, lifetime alcohol and lifetime drug misuse, unemployment, and no current partner;
• exclusively basic symptoms: family history of mental disorders, younger age, unemployment, no current partner, and single marital status;
• ultra-high risk and basic symptoms combined: family history of mental disorders, female sex, less school education, both lifetime and current alcohol and drug misuse, younger age, and lifetime trauma.

No variable exclusively predicted the presence of APS/BIPS alone. Urbanicity and both migrant and minority status were unrelated to psychosis-risk symptoms (online Supplementary Table S4).

### Risk factors for presence of psychosis-risk criteria

The presence of any psychosis-risk criterion was predicted by a family history of mental disorder, lifetime drug misuse, lifetime traumatic event, and urbanicity. Age, sex, minority or migrant status, school education, unemployment, single marital status, current partner, lifetime and current alcohol misuse, or current drug misuse did not predict psychosis-risk criteria (Table 5). All four main predictors were selected for and remained in the stepwise model, although urbanicity exerted an extremely low effect (OR: 1.000, 95% CI: 1.000–1.001) (Table 5).

**Table 5.** Association of presence of any psychosis-risk criterion with predictors described for psychotic-like experiences, assessed by questionnaires or fully-standardized lay-person interviews for psychotic symptoms in the community (Linscott & van Os, 2013)

	*β*	Standard error	Wald (df = 1)	*p* value	Odds ratio	95% lower	CI upper
Results of univariate logistic regression analyses							
Age^a^	−0.023	0.016	2.031	0.154	0.977	0.946	1.009
Male sex^a^	−0.419	0.255	2.699	0.100	0.658	0.399	1.084
School education^a^	−0.089	0.171	0.271	0.602	0.915	0.655	1.279
Current unemployment^a^	−0.758	0.606	1.565	0.211	0.469	0.143	1.536
Migrant status^a^	0.225	0.473	0.227	0.634	1.252	0.496	3.162
Minority status^a^	0.499	1.028	0.236	0.627	1.647	0.220	12.345
Single marital status^a^	0.494	0.269	3.363	0.067	1.638	0.967	2.776
No current partner^a^	−0.453	0.262	2.996	0.083	0.636	0.380	1.062
Family history of mental disorder^b^	*0.783*	*0.258*	*9.228*	*0.002*	*2.188*	*1.320*	*3.626*
Lifetime traumatic event^b^	*1.077*	*0.296*	*13.249*	*<0.001*	*2.935*	*1.644*	*5.241*
Lifetime alcohol misuse^a^	0.498	0.526	0.895	0.344	1.645	0.587	4.614
Current alcohol misuse^a^	0.959	0.740	1.677	0.195	2.608	0.611	11.124
Lifetime drug misuse^b^	*1.153*	*0.319*	*13.031*	*<0.001*	*3.169*	*1.694*	*5.927*
Current drug misuse^a^	0.758	0.736	1.060	0.303	2.134	0.504	9.035
Population density (person/km^2^)^b^	*0.000*	*0.000*	*7.427*	*0.006*	*1.000*	*1.000*	*1.001*
Results of stepwise logistic regression analyses (Wald method, forward and backward)^c^							
Positive family history	0.697	0.260	7.175	0.007	2.007	1.206	3.341
Lifetime drug misuse	0.938	0.328	8.189	0.004	2.555	1.344	4.858
Lifetime traumatic event	0.884	0.308	8.257	0.004	2.421	1.325	4.424
Population density	0.000	0.000	5.595	0.018	1.000	1.000	1.001

CI, confidence interval of odds ratio.

a All models were non-significant with a goodness-of-fit of χ^2^_(1)_⩽3.521, *p*>0.061.

b All models were significant with a goodness-of-fit of χ^2^_(1)_⩾6.650, *p*<0.010.

c The model was highly significant with a goodness-of-fit of χ^2^_(4)_ = 31 175, *p*<0.001; correct classification of risk-negative cases: 100%, correct classification of risk-positive cases: 0%.

Significant variables at a *p*-level of 5% in univariate analyses are displayed in *Italics*.

## Discussion

An efficient indicated prevention of mental disorders requires valid risk criteria that work in both clinical and community samples. In the case of psychotic disorders, risk criteria are available that were recommended for use in clinical samples but not for use in the community for lack of knowledge about their prevalence and clinical relevance in non-clinical settings (Schultze-Lutter *et al.* 2015*b*).

### Prevalence of psychosis-risk symptoms and criteria

#### Ultra-high risk symptoms and criteria

Community studies of psychotic-like experiences found a median prevalence of 7.2% (range: 0.5%–47.2%) with higher rates in convenience, non-dispersed, and smaller samples (Linscott & van Os, 2013). Psychotic-like experiences were frequently assumed to resemble APS or even the APS criterion (Schultze-Lutter *et al.* 2011, 2014*b*), although their validity was not sufficiently assured and the onset and frequency requirements of the APS criterion were commonly not assessed. Thus, recent reviews and studies indicated significant overestimation of APS/BIPS by and little content validity of questionnaire-assessed psychotic-like experiences compared with the gold standard of the assessment of APS/BIPS in a (clinical) interview (Linscott & van Os, 2013; Schultze-Lutter *et al.* 2014*b*). To avoid such an overestimation, we assessed a large randomly selected, dispersed, representative community sample of 16- to 40-year-old individuals with semi-structured clinical interviews specifically designed for the assessment of psychosis-risk symptoms and criteria. Hence, unsurprisingly, the 6% prevalence rate of current APS/BIPS that were reported as a change from earlier thought contents and perceptions as well as the 0.6% prevalence rate of APS/BIPS criteria were below the reported median rate of psychotic-like experiences. Furthermore, the prevalence rate of current APS/BIPS was in line with the 5.8% lifetime prevalence of psychotic symptoms reported in the World Mental Health Survey (McGrath *et al.* 2016).

#### Basic symptoms and basic symptom criteria

For basic symptoms and related criteria, community studies have not been performed. Thus, their prevalence rates of almost 10% for any current basic symptom and 2% for any basic symptom criterion, mainly by COPER, cannot be compared with other findings. The higher prevalence of COPER compared with COGDIS, however, is in line with findings in clinical samples that found COPER to be more sensitive and COGDIS more specific (Schultze-Lutter *et al.* 2012).

#### Any psychosis-risk symptom and criterion

Overall, 14% of participants reported current psychosis-risk symptoms as a change in mental processes or experiences. Psychosis-risk symptoms occurred mainly infrequent; consequently, psychosis-risk criteria were met by a mere 2.4%, reaching as low as 1.1% if only psychosis-risk criteria recommended by the European Psychiatric Association were considered, i.e. APS, BIPS, and/or COGDIS (Schultze-Lutter *et al.* 2015*b*). Thus, if added to the 1.4% rate of participants excluded for past or present psychoses (Michel *et al.* 2016), the point-prevalence of participants considered at clinical high-risk for psychosis is as high—or even slightly lower—as that expected from the reported lifetime prevalence of any non-organic psychotic disorder of 3.5% (Perälä *et al.* 2007).

### Clinical significance of psychosis-risk symptoms and criteria

Irrespective of their potential association with the future development of a psychotic disorder, the presence of any psychosis-risk symptom and, more strongly, of any psychosis-risk criterion, was associated with a significant 4- to 17-fold increased odds of current mental disorder and current functional deficit, respectively, indicating their clinical relevance. Expectantly, the association of the type of psychosis-risk symptoms and criteria with mental illness and functional deficits demonstrated a stepwise effect. The combined presence of ultra-high risk and basic symptoms and criteria were the most strongly related and, with one exception, basic symptoms and related criteria were significantly but least strongly associated with mental disorder and functional impairment. Interestingly, the association of psychosis-risk symptoms and criteria with a functional deficit was commonly stronger than that with a mental disorder, indicating that psychosis-risk symptoms and criteria are not merely a manifestation of mental ill-health.

### Risk factors for presence of any psychosis-risk symptom and criterion

In community studies of psychotic-like experiences, risk factors for their presence were younger age, minority status, lower income, single marital status, substance misuse, exposure to stressful or traumatic events, and family history of mental illness, while there was no evidence that sex, migrant status, education, unemployment, or urbanicity increased odds of their report (Linscott & van Os, 2013). All but minority status were also related to the presence of psychosis-risk symptoms in our study, which was additionally predicted by lower education and unemployment. However, ORs were commonly small, ranging from 1.35 for single marital status to 2.64 for current drug misuse in univariate analyses and were below 2 in the multivariate model.

Moderate influences of sex, age, and, largely explained by age, education years on APS/BIPS have also been reported from a Swiss patient sample (Theodoridou *et al.* 2017). Moreover, a recent review on the impact of cannabis as the most commonly used drug reinforced its role in the development of psychotic and schizotypal symptoms, with family history and traumatic events likely increasing sensitivity to cannabis (Løberg *et al.* 2014). Supporting these findings, the presence of any psychosis-risk criterion was related to a history of a first- or second-degree relative with mental disorder, lifetime drug misuse, and lifetime traumatic event. In this, the effect of a positive family history was primarily driven by the reports of depressive disorders in family members (in 37% of individuals with a psychosis-risk criterion). Reported psychotic disorders of relatives were not significantly related to psychosis-risk criteria (in 6% of individuals with a psychosis-risk criterion) or any type of psychosis-risk symptoms. A higher rate of family members with a depressive disorder (57%) compared with a psychotic disorder (11%) was also reported in an adolescent ultra-high risk sample (Simeonova *et al.* 2015).

In line with the findings on psychotic-like experiences (Linscott & van Os, 2013) but contrary to the findings on psychosis (Vassos *et al.* 2012), the statistically significant effect of urbanicity on psychosis-risk criteria was negligible in our semi-rural recruitment area with Nidau (*n* = 28) showing the highest population density of 4480 individuals/km^2^ and Ostermundigen (n = 26) the second highest (2643 individuals/km^2^). By comparison, Greater London's population density is reported as 5518 individuals/km^2^ (source: Wikipedia). Thus, a stronger effect of urbanicity might have been missed due to the lack of high urbanicity levels.

### Differential risk factors for presence of ultra-high risk or basic symptom and related criteria

When symptoms of the ultra-high risk and basic symptom approach were considered separately, the moderators differed greatly. In line with our expectations, younger age was selectively related to basic symptoms, supporting the earlier notion that APS might be more common but less clinically relevant and predictive of psychosis below the age of 15/16 years (Cornblatt *et al.* 2015; Schimmelmann *et al.* 2015). The age effect on the basic symptoms groups, however, might indicate a potentially higher age threshold for basic symptoms that still works within this sample's age range, possibly because of the brain maturation processes still ongoing in the younger age segment (Schultze-Lutter *et al.* 2012, 2016).

The likelihood of the presence of APS/BIPS was selectively increased by reports of traumatic events, as well as lifetime misuse of either alcohol or drugs. This supports models of APS/BIPS relating their evolution to dysfunctional coping with stressors, including the development of inadequate explanatory models (Bentall *et al.* 2007; Gebhardt *et al.* 2008).

### Strengths and limitations

To our knowledge, this is the first study to examine validly the prevalence of all relevant psychosis-risk symptoms and criteria in a large random community sample of the age segment at highest risk of psychosis (Kirkbride *et al.* 2006) in a manner comparable with clinical assessment. Prior to commencing the study, we found that telephone interviews were a reliable method of validly assessing psychosis-risk symptoms (Michel *et al.* 2014). Hence, telephone interviews were selected over face-to-face interviews for their lower costs and assumed better response rate (e.g. less time spent travelling for interviewers and participants). However, the availability of telephone numbers was slightly associated with older age; yet, this selection bias was so small that it did not introduce a significant difference in the distribution of participants across age groups. The potential age bias is therefore at most a negligible limitation of our study. Thus, at a sufficiently large response rate of 63% and with no meaningful difference between participants and the population statistics, our sample can be regarded as representative of the young adult population of the Canton of Bern.

We had assumed the failure of contact attempts of >100 as indicative of prolonged absence and, consequently, ineligibility. This could have introduced a selection bias, as psychosis-risk symptoms might be more prevalent in hard-to-reach individuals. However, such a bias is unlikely as the number of attempts before the interview was unrelated to the presence of psychosis-risk symptoms (OR: 0.994; 95% CI: 0.986–1.003).

Beside the above-discussed possible area bias on the effect of urbanicity, a language-related bias toward not including individuals with migration/minority status was detected that our study shares with several mental health studies (Brown *et al.* 2014). This was despite our efforts to minimise this bias by conducting interviews in four different languages including those commonly spoken in African and South-American countries. This bias might have led to an underestimation of the influence of minority status in particular that was related to a 3–6 times increased likelihood of presenting with psychosis (Bosqui *et al.* 2014).

Different effects of potential moderators might have also been observed had we analysed them with respect to lifetime and not only current psychosis-risk symptoms. This focus was selected, however, to avoid the probable impact of a combined recognition and recollection bias in disfavour of basic symptoms described for clinical samples (Schultze-Lutter *et al.* 2010).

### Implications

Within these minor constraints, the results of this unique representative community study demonstrate that the broad implementation of psychosis-risk criteria, e.g. in primary care or counselling services, will not result in pathologising common non-ill experiences in young adults. Furthermore, the indicated clinical relevance of both psychosis-risk symptoms and criteria, in particular the combined presence of ultra-high risk and basic symptoms, as well as their predominant association with functional impairment, reinforce the need to consider these symptoms in treatment plans. Thus, beyond any potential risk of developing psychosis, clinicians should probe for psychosis-risk symptoms, especially in young adults and patients with a positive family history of mental disorders and history of trauma and/or of substance use. Greater insight into the longitudinal relationship of psychosis-risk symptoms and criteria to the development of frank psychosis will be gained from future follow-ups.

## Supplementary material

For supplementary material accompanying this paper visit http://dx.doi.org/10.1017/S0033291717002586. click here to view supplementary material
