# Supplementary material for: Prevalence and clinical relevance of interview-assessed psychosis-risk symptoms in the young adult community
Source: Psychol Med. 2017 Sep 11;48(7):1167–78. doi: 10.1017/S0033291717002586 (PMC6088777; doi:10.1017/S0033291717002586)
Supplement: Supplementary file 1 [file S0033291717002586sup001.pdf]

Supplementary Material to:
Prevalence and clinical relevance of interview-assessed psychosis risk symptoms in the young adult community.

F. Schultze-Lutter; C. Michel; S. Ruhrmann; B.G. Schimmelmann

Contents

- **Supplementary Text S1:** A brief introduction into ultra-high risk and basic symptom criteria and symptoms
    - **Table S1.** Ultra-high risk and basic symptom criteria
- **Supplementary Table S2:** Reasons for refusal
- **Supplementary Table S3:** Sociodemographic and clinical characteristics of persons with a complete interview
- **Supplementary Table S4:** Association of the different types of current non-trait-like CHR symptoms with predictors of psychotic-like experiences
- **References Supplementary Material**

Supplementary Text S1: A brief introduction into ultra-high risk and basic symptom criteria and symptoms

Target period of risk detection

Ultra-high risk criteria (Supplementary Table S1) were originally developed with the explicit aim of detecting an imminent risk for psychoses, i.e., persons at risk for developing a first-episode within the next 12 months (Phillips *et al*. 2000). In contrast, basic symptom criteria (Supplementary Table S1) were developed to detect the emerging psychotic disorder as early as possible, desirably before the onset of functional decline (Schultze-Lutter, 2016; Schultze-Lutter *et al*. 2016).

Supplementary Table S1. The symptom-related criteria of the ultra-high risk criteria according to the Structured Interview of Prodromal Syndromes (SIPS; McGlashan *et al*. 2010) and the basic symptom criteria according to the Schizophrenia Proneness Instrument, Adult version (SPI-A; Schultze-Lutter *et al*. 2007)

Ultra-high risk criterion ‘Attenuated Psychotic Symptoms’ (APS)

➲ At least any 1 of the following 5 items with a SIPS score of ‘3’ to ‘5’:

- ***unusual thought content / non-paranoid, non-grandiose delusional ideas (P1)*** not held with full conviction, including magical ideation, non-paranoid ideas of reference not immediately rectified by cognition, and attenuated “Ich-Störungen”
- ***suspiciousness / persecutory ideas (P2)*** not held with full conviction
- ***grandiose ideas (P3)*** of special powers or missions not held with full conviction
- ***perceptual abnormalities / hallucinations (P4)*** with remaining insight in their abnormal nature, incl. schizotypal phenomena such as sensing the presence of something/someone, perceiving moving shadows in the periphery of the visual field or unusual bodily perceptions
- ***disorganized communication (P5)*** and speech that is still comprehensible and responds to structuring in the interview

➲ First occurrence or worsening (in terms of an increase in conviction / loss of insight and/or of an increased impact on behaviour) within the past 12 months.

➲ At least weekly occurrence within past month.

Ultra-high risk criterion ‘Brief Intermittent Psychotic Symptoms’ (BIPS)

➲ At least any 1 of the above 5 items (P1-P5) with a SIPS score of ‘6’, i.e. temporarily held with full conviction or with complete lack of insight

➲ Psychotic level of intensity, i.e., a score of ‘6’ was reached within past 3 months.

➲ At least present for several minutes per day at a frequency of at least once per month.

Basic symptom criterion ‘Cognitive-Perceptive Basic Symptoms’ (COPER)

➲ At least any 1 of the following 10 basic symptoms:

- ***thought interference (D9)***: Irrelevant, emotionally neutral thoughts with no special meaning and no association with the intended thought are intruding on and disturbing the young person’s train of thought, without it being lost.
- ***thought perseveration (C2)***: A kind of thought interference in that intruding emotionally neutral and irrelevant thoughts or images occur not just once but repeatedly.
- ***thought pressure (D10)***: A self-reported ‘chaos’ of thoughts in that successively occurring thoughts are not linked by any common thread, and are completely unrelated to each other or to the young person’s intended line of thought.
- ***thought blockages (D15)***: Sudden interruption in the flow of thoughts, or experiences of the mind suddenly going blank, of a fading (slipping) of thoughts or of losing the thread of thoughts, with the original topic being recalled subsequently or lost completely.
- ***disturbance of receptive speech (D11)***: A disturbance in the understanding of simple everyday words. When reading or listening to others, the young person struggles to comprehend the meaning of words, word sequences or sentences, even if the young person concentrates on the text or speech and has perceived it accurately.

- ***decreased ability to discriminate between ideas and perception, fantasy and true memories (B2)***: A self-recognized difficulty in locating the source of an experience/memory (external vs. internal mental) that results in an inability to immediately distinguish between imaginations and perception, or pure fantasy and true memories.
- ***unstable ideas of reference (B2)***: Subjective, subclinical experiences of self-reference for that no explanation outside own mental processes are sought, and that is immediately overcome.
- ***derealisation (O8)***: A change in how one relates emotionally to the environment, which is experienced commonly as an estrangement and detachment from the visual world, or rarely as an increased emotional affinity for the environment.
- ***visual perception disturbances (O4)*** (excl. blurred vision and hypersensitivity to light): Misperceptions of aspects of the visual field while the young person is fully aware of their true appearance and, therefore, attributes his or her misperception to a problem with eye sight or mental processes.
- ***acoustic perception disturbances (O5)*** (excl. hypersensitivity to sounds/noises): Misperceptions of acoustic stimuli while the young person is fully aware of the true sound and, therefore, tends to attribute his or her misperception to a problem with hearing or mental processes.

➲ First occurrence or significant increase in frequency ≥12 months ago

➲ Occurrence of at least ‘several times in a month or weekly’ within the past 3 months, i.e. a SPI-A score of at least ‘3’.

Basic symptom criterion ‘Cognitive Disturbances’ (COGDIS)

➲ At least any 2 of the following 9 basic symptoms:

- ***inability to divide attention (B1)***: A difficulty in dealing with demands that involve more than one sensory modality at a time and thus does not concern demands that would require quick switching of attention.
- ***captivation of attention by details of the visual field (O7)***: Domination of the visual field by a random single aspect of it that captures the young person’s whole attention, impedes paying attention to other aspects and causes difficulties in turning away from it.
- ***thought interference*** (see COPER)
- ***thought pressure*** (see COPER)
- ***thought blockages*** (see COPER)
- ***disturbance of receptive speech*** (see COPER)
- ***disturbance of expressive speech (C5)***: A subjective difficulty in verbal fluency and clarity of expression, with words required to express simple ideas being not forthcoming or delayed.
- ***disturbances of abstract thinking (O3)***: Deficits in the comprehension of any kind of abstract, figurative or symbolic phrases or content, as well as the phenomena of ‘concretism’ (a limitation of the ability to go beyond the literal meaning of words, sentences or phrases).captivation of attention by details of the visual field (O2)
- ***unstable ideas of reference*** (see COPER)

➲ Occurrence of at least ‘several times in a month or weekly’ within the past 3 months, i.e. a SPI-A score of at least ‘3’.

Note: A general requirement of basic symptoms is their novelty, i.e., their report as a disruption in a person’s “normal” self. Self-recognized aberrations in mental processes that have always been present in the same frequency, i.e., in a trait-like manner, can be rated in SPI-A (rating of “7”) but are not accounted for as basic symptoms in the strict sense and, consequently, do not contribute to basic symptoms criteria. More in-depth definitions of basic symptoms as well as example statements of patients and example questions for their assessment are provided in the SPI-A, orderable at www.fioriti.it.

Definition of basic symptoms

Basic symptoms were conceptualised as the earliest primarily self-experienced psychopathological correlates of the physiological disturbances of information processing underlying the development of psychosis that develops on the basis of and partly in reaction to them (Schultze-Lutter *et al*. 2016). By

definition, basic symptoms differ from what patients’ consider to be their ‘normal’ mental self, and thus, are distinct from trait-like schizotypy features considered as part of the ‘normal’ self (Schultze-Lutter, 2009). Furthermore, basic symptoms remain predominately private and are rarely observable to others, although patients’ self-initiated coping strategies (including avoidance strategies and social withdrawal) in response to their basic symptoms may be observable, e.g., as negative symptoms.

Phenomenological differences between basic and ultra-high risk / schizotypal symptoms

For their spontaneous, immediate recognition by patients as disturbances of their own (mental) processes, basic symptoms are also distinct from the symptoms that define the ultra-high risk criteria (i.e., attenuated psychotic symptoms (APS) or brief intermittent psychotic symptoms (BIPS)) and from frank psychotic symptoms, in which reality testing is disturbed at least to some degree. Within the basic symptoms concept, (attenuated) psychotic symptoms are considered to arise from basic symptoms when everyday situations and demands overstrain patients’ already pathologically vulnerable information processing capacity. Thus, given the salutary environmental and personal conditions (e.g., a supportive social network; good social, problem solving, and coping skills; or high self-efficacy), basic symptoms can be counterbalanced as long as their number and/or severity do not overextend protective factors and patients’ resilience (Schultze-Lutter, 2009; Schultze-Lutter, 2016).

Basic symptoms are phenomenologically distinct from mental states that an individual would consider being his or her ‘normal’ self. Thus, the novelty of basic symptoms distinguishes them from the subtle long-standing disturbances that present as traits in those at genetic high-risk (Parnas & Carter, 2002; Jones, 2002). In addition, basic symptoms are phenomenologically distinct from APS, BIPS and frank psychotic symptoms because they are not necessarily observable by others in terms of odd thinking, disturbed speech, or formal thought disorder. BS are subtle and subjective, in the sense that they rarely affect observable speech, thought content or behaviour, unlike the more typical psychotic or schizotypal phenomena of magical thinking, ideas of reference, paranoid ideation, suspiciousness, delusions, and ‘Ich-Störungen’ (i.e., thought broadcasting; insertion, withdrawal, and delusion of alien control). Furthermore, they may be distinguished from negative symptoms in that they are not observable functional deficits (Parnas *et al.* 2005) but remain completely in the subjective world of the individual experiencing them. Commonly, BS are not a part of the definition of mental disorders, although basic symptoms that are not part of COGDIS or COPER can be reported in non-psychotic mental disorders (Klosterkötter *et al.* 1996).

Differences between ultra-high risk and schizotypal symptoms

In contrast to the basic symptoms criteria, the ultra-high risk criteria and symptoms were mainly modelled on the subthreshold psychotic-like experiences as defined by Chapman and colleagues (Chapman & Chapman, 1980) and positive features of schizotypal personality disorder (Debbané *et al.* 2015). Nevertheless, ultra-high risk symptoms differ from the trait-like features of a schizotypal (personality) disorder as they have a defined time of onset or worsening by definition and for the focus of indicated prevention on the first signs of the emerging disorder. Besides this partly phenomenological overlap of ultra-high risk criteria with features of a schizotypal (personality) disorder and, to a lesser degree, of a paranoid personality disorder (Schultze-Lutter *et al.* 2012), APS are not part the definition of non-psychotic axis-I and other axis-II disorders.

**Supplementary Table S2:** Reasons for refusal (N=890[a]), multiple answers possible	
Lack of interest in topic, No. (%)	498 (56.0)
Lack of time, No. (%)	423 (47.5)
Assessment of too intimate data expected, No. (%)	135 (15.2)
Interview too long, No. (%)	132 (14.8)
No immediate personal gain, No. (%)	53 (6.0)
Uncomfortable to talk on the phone, but would be willing to participate alternatively, No. (%)<br>• in a face-to-face interview at home (n=2)<br>• in a face-to-face interview at the clinic (n=2)<br>• via questionnaire (n=8)<br>• some other way, e.g. via SMS, email (n=32)	44 (4.9)
Unimportant topic, No. (%)	43 (4.8)
Insufficient trust in data security and anonymity, No. (%)	38 (4.3)
Avoidance of contact with psychiatry, No. (%)	10 (1.1)
Bad experiences with psychiatry, No. (%)	2 (0.2)

[a] Additional 353 refusers gave no reason for refusal

Supplementary Table S3: Sociodemographic and clinical characteristics of persons with a complete interview (N=2683)

**Age** in years (mean±standard deviation)		30.2±7.6
**Age ranges** (%)		
	16-20 years	16.1
	21-25 years	16.5
	26-30 years	11.5
	31-35 years	20.9
	36-40 years	34.9
**Sex**; % male		54.0
**Nationality**; % Swiss		93.6
**Minority status**; % yes[a,c]		1.0
**Marital status** (%)		
	Single	55.9
	Married/extra-marital cohabitation	40.4
	Separated/divorced/widowed	3.7
**Current partnership**; % yes[a]		72.2
**Highest education** (%)[a,d]		
	ISCED 1	0.2
	ISCED 2	4.0
	ISCED 3	7.4
	ISCED 4	0.7
	ISCED 5	55.3
	ISCED 7	31.2
	ISCED 8	1.3
**Highest school level** (%)[a,d]		
	ISCED 1	1.3
	ISCED 2	59.7
	ISCED 3 (35)	39.0
**Current occupation** (%)[a]		
	Unemployed	2.3
	Sheltered employment	0.3
	Only temporarily employed	1.1
	Normal employment / education	96.3
**Current living condition** (%)[a]		
	Alone	12.2
	With partner (and children)	53.4
	With children	1.7
	With parent(s)	27.1
	With other relatives	0.4
	With other persons/friends	5.1
	In foster family / home	0.1
**Population density,** mean±standard deviation (range)[a]		803±916 (1-4480)

Supplementary Table S4 cont.

**Family history**[a,e] (%)		
	Psychotic disorder	2.7
	Affective disorder	23.0
	Anxiety disorder	1.1
	Obsessive-compulsive disorder (OCD)	0.2
	Substance-use disorder	3.2
	Other or unknown but very likely mental disorder[f]	12.9
**Alcohol misuse** (%)[a,g]		
	lifetime	4.0
	current	1.3
**Illegal drug misuse** (%)[a,g]		
	lifetime	7.8
	current	1.5
**Current non-psychotic axis-I disorder** (%)[a,g]		13.1
	Affective disorder	4.2
	Anxiety disorder	9.4
	Obsessive-compulsive disorder	0.8
	Eating disorder	0.4
	Somatoform disorder	1.0
	Posttraumatic stress disorder	0.7
**SOFAS score,** mean±standard deviation (range)[a]		85.3±7.1(39-99)
**Functional deficit**; % yes[a,h]		5.5
**Lifetime traumatic event**; % yes[a,i]		10.5

[a] Not compared to the Bern statistics, because age group-specific data were not provided.

[b] Effect sizes were Cohen’s d for the t-test and the effect size index, w, for the 1-dimensional χ^2-tests. For Cohen’s d, d=0.2 equals a small effect, d=0.5 a medium effect, and d=0.8 a large effect; for the effect size index w, w=0.1 equals a small effect, w=0.3 a medium effect, and w=0.5 a large effect.

[c] estimated by a nationality of a non-Western country with many non-Caucasian inhabitants, esp. South America, Asia and Africa

[d] ISCED: International Standard Classification of Education, 2011 revision (http://www.uis.unesco.org/Education/Pages/international-standard-classification-of-education.aspx). Description of main categories: 2=lower secondary education; 3=upper secondary education; 5= short-cycle tertiary education, and 7=Master or equivalent.

[e] in 1st- or 2nd-degree biological relatives; known as well as very likely main disorder, multiple relatives possible, maximum reported number of affected relatives was n=5

[f] “Other” includes frequent report of “burn-out” as well as of severe developmental and personality disorders, “unknown but very likely” includes reports of relatives who had received help for largely unspecified mental problems or were known in the family for mental problems associated with behavioral abnormalities / functional impairments

[g] according to the M.I.N.I.

[h] defined by a Social and Occupational Functioning Assessment Scale (SOFAS) score of 70 or less.

[i] estimated by a positive answer to the M.I.N.I.-screening question for a posttraumatic stress disorder

Supplementary Table S4: Association of the different types of current non-trait-like psychosis-risk symptoms with predictors of psychotic-like experiences, assessed by questionnaires or fully-standardized lay-person interviews for psychotic symptoms in the community (Linscott & van Os, 2013). Results of significant nominal regression models with “no psychosis-risk symptom” as reference group.

		β	standard error	Wald (df=1)	p-value	odds ratio	95% CI lower	95% CI upper
Age<br>GoF: $\chi^2_{(3)}$=17.049, p=0.001	only basic symptoms	*-0.034*	*0.009*	*13.234*	*<0.001*	*0.967*	*0.950*	*0.985*
	only APS/BIPS	-0.007	0.013	0.327	0.568	0.993	0.967	1.018
	both	*-0.040*	*0.019*	*4.494*	*0.034*	*0.961*	*0.926*	*0.997*
Sex (female)<br>GoF: $\chi^2_{(3)}$=9.287, p=0.026	only basic symptoms	0.035	0.143	0.059	0.809	1.035	0.783	1.369
	only APS/BIPS	0.183	0.199	0.848	0.357	1.201	0.813	1.772
	both	*0.876*	*0.309*	*8.038*	*0.005*	*2.402*	*1.311*	*4.401*
School education<br>GoF: $\chi^2_{(3)}$=11.703, p=0.008	only basic symptoms	-0.038	0.095	0.163	0.687	0.962	0.799	1.159
	only APS/BIPS	-0.268	0.138	3.749	0.053	0.765	0.583	1.003
	both	*-0.594*	*0.222*	*7.168*	*0.007*	*0.552*	*0.357*	*0.853*
Current unemployment<br>GoF: $\chi^2_{(3)}$=8.888, p=0.031	only basic symptoms	*0.872*	*0.356*	*5.989*	*0.014*	*2.392*	*1.190*	*4.811*
	only APS/BIPS	*1.084*	*0.446*	*5.911*	*0.015*	*2.957*	*1.234*	*7.086*
	both	0.026	1.021	0.001	0.979	1.027	0.139	7.599
Single marital status<br>GoF: $\chi^2_{(3)}$=9.691, p=0.021	only basic symptoms	*0.417*	*0.148*	*7.907*	*0.005*	*1.518*	*1.135*	*2.031*
	only APS/BIPS	0.034	0.200	0.029	0.865	1.035	0.699	1.532
	both	0.407	0.305	1.788	0.181	1.503	0.827	2.731
No current partner<br>GoF: $\chi^2_{(3)}$=12.787, p=0.005	only basic symptoms	*0.384*	*0.150*	*6.524*	*0.011*	*1.469*	*1.094*	*1.972*
	only APS/BIPS	*0.474*	*0.207*	*5.262*	*0.022*	*1.607*	*1.072*	*2.411*
	both	0.505	0.302	2.797	0.094	1.657	0.917	2.993

Supplementary Table S5 cont.

		β	standard error	Wald (df=1)	p-value	odds ratio	95% CI lower	95% CI upper
Family history of mental disorders GoF: $\chi^2_{(3)}$=24.733, p<0.001	only basic symptoms	*0.331*	*0.144*	*5.293*	*0.021*	*1.392*	*1.050*	*1.846*
	only APS/BIPS	*0.508*	*1.99*	*6.512*	*0.011*	*1.662*	*1.125*	*2.455*
	both	*1.146*	*0.305*	*14.140*	*<0.001*	*3.147*	*1.731*	*5.720*
Lifetime traumatic event GoF: $\chi^2_{(3)}$=17.095, p=0.001	only basic symptoms	0.379	0.212	3.210	0.073	1.461	0.965	2.213
	only APS/BIPS	*0.716*	*0.263*	*7.426*	*0.006*	*2.047*	*1.223*	*3.426*
	both	*1.139*	*0.341*	*11.179*	*0.001*	*3.124*	*1.602*	*6.091*
Lifetime alcohol misuse GoF: $\chi^2_{(3)}$=9.366, p=0.025	only basic symptoms	0.278	0.343	0.659	0.417	1.321	0.675	2.586
	only APS/BIPS	*0.926*	*0.366*	*6.396*	*0.011*	*2.524*	*1.232*	*5.174*
	both	*1.152*	*0.486*	*5.623*	*0.018*	*3.164*	*1.221*	*8.196*
Current alcohol misuse GoF: $\chi^2_{(3)}$=5.615, p=0.132	only basic symptoms	0.214	0.614	0.122	0.727	1.239	0.372	4.127
	only APS/BIPS	0.526	0.741	0.504	0.478	1.692	0.396	7.233
	both	*1.769*	*0.628*	*7.932*	*0.005*	*5.864*	*1.712*	*20.082*
Lifetime drug misuse GoF: $\chi^2_{(3)}$=17.856, p<0.001	only basic symptoms	0.247	0.254	0.951	0.330	1.281	0.779	2.105
	only APS/BIPS	*0.999*	*0.271*	*13.571*	*<0.001*	*2.716*	*1.596*	*4.621*
	both	*1.120*	*0.379*	*8.744*	*0.003*	*3.064*	*1.459*	*6.436*
Current drug misuse GoF: $\chi^2_{(3)}$=8.024, p=0.046	only basic symptoms	0.811	0.454	3.188	0.074	2.250	0.924	5.481
	only APS/BIPS	0.830	0.615	1.824	0.177	2.294	0.688	7.654
	both	*1.658*	*0.625*	*7.043*	*0.008*	*5.251*	*1.543*	*17.869*

CI: confidence interval of odds ratio

GoF: goodness-of-fit; APS: attenuated psychotic symptom; BIPS: brief intermittent psychotic symptom.

Significant variables at a p-level of 5% in univariate analyses are displayed in *Italics*.

References Supplementary Material

1. **Chapman LJ, Chapman JP** (1996). Scales for rating psychotic and psychotic-like experiences as continua. *Schizophrenia Bulletin* **6**, 477-489.
2. **Debbané M, Eliez S, Badoud D, Conus P, Flückiger R, Schultze-Lutter F** (2015). Developing psychosis and its risk states through the lens of schizotypy. *Schizophrenia Bulletin* **41**, 396-407.
3. **Jones PB** (2002). Risk factors for schizophrenia in childhood and youth. In *Risk and Protective Factors in Schizophrenia. Towards a Conceptual Model of the Disease Process* (ed. H. Häfner), pp. 141-162. Steinkopff: Darmstadt.
4. **Klosterkötter J, Ebel H, Schultze-Lutter F, Steinmeyer EM** (1996). Diagnostic validity of basic symptoms. *European Archives of Psychiatry and Clinical Neuroscience* **246**, 147-154.
5. **Linscott RJ, van Os J** (2013). An updated and conservative systematic review and meta-analysis of epidemiological evidence on psychotic experiences in children and adults: on the pathway from proneness to persistence to dimensional expression across mental disorders. *Psychological Medicine* **43**,1133-1149.
6. **McGlashan T, Walsh B, Woods S** (2010). *The Psychosis-Risk Syndrome. Handbook for Diagnosis and Follow-Up*. Oxford University Press: New York.
7. **Parnas J, Carter JW** (2002). High-risk studies and neurodevelopmental hypothesis. In *Risk and Protective Factors in Schizophrenia. Towards a Conceptual Model of the Disease Process* (ed. H. Häfner), pp. 71-82. Steinkopff: Darmstadt.
8. **Parnas J, Handest P, Jansson L, Saebye D** (2005). Anomalous subjective experience among first-admitted schizophrenia spectrum patients: empirical investigation. *Psychopathology* **38**, 259-267.
9. **Phillips LJ, Yung A, McGorry PD** (2000). Identification of young people at risk of psychosis: validation of the Personal Assessment and Crisis Evaluation Clinic intake criteria. *Australian and New Zealand Journal of Psychiatry* **34**, 164-169.
10. **Schultze-Lutter F** (2009). Subjective symptoms of schizophrenia in research and the clinic: the basic symptom concept. *Schizophrenia Bulletin* **35**, 5-8.
11. **Schultze-Lutter F** (2016). First signs of emerging psychosis. In *Early Detection and Intervention in Psychosis - State of the Art and Future Perspectives; Key Issues in Mental Health* (ed. A. Riecher-Rössler A and P.D. McGorry), pp. 29-41. Karger: Basel.
12. **Schultze-Lutter F, Addington J, Ruhrmann S, Klosterkötter J** (2007). *Schizophrenia Proneness Instrument, Adult version (SPI-A)*. Giovanni Fioriti Editore s.r.l.: Rome.
13. **Schultze-Lutter F, Debbané M, Theodoridou A, Wood SJ, Raballo A, Michel C, Schmidt SJ, Kindler J, Ruhrmann S, Uhlhaas PJ** (2016). Revisiting the Basic Symptom Concept: Toward Translating Risk Symptoms for Psychosis into Neurobiological Targets. *Frontiers in Psychiatry* **7**, 9.
14. **Schultze-Lutter F, Klosterkötter J, Michel C, Winkler K, Ruhrmann S** (2012). Personality disorders and accentuations in at-risk persons with and without conversion to first-episode psychosis. *Early Intervention in Psychiatry* **6,** 389-398.
